# Mild and Asymptomatic Covid-19 Infections: Implications for Maternal, Fetal, and Reproductive Health

**DOI:** 10.3389/frph.2020.00001

**Published:** 2020-06-16

**Authors:** Bei Sun, John Yeh

**Affiliations:** ^1^Sackler School of Medicine, New York State/American Program of Tel Aviv University, Tel Aviv, Israel; ^2^Department of Obstetrics & Gynecology, University of Massachusetts Medical School, UMass Memorial Medical Center, Worcester, MA, United States

**Keywords:** Covid-19, pregnancy, asymptomatic women, placenta, fetus, health risk

## Introduction

As of May 2020, more than five million people worldwide tested positive for SARS-CoV-2, among which around 80% display mild or no symptoms (1). There are currently around 4 million people worldwide in this category. According to these and other statistics, asymptomatic and mildly symptomatic infected pregnant women outnumber those infected women requiring hospitalization. For example, in a report from New York City about 43 pregnant women who tested positive for SARS-CoV-2 over the course of a 2 week period in March, 2020, the authors found that 86% of COVID-19 pregnant patients presented with mild or no viral-associated symptoms (2). Reports from China and Europe corroborate that asymptomatic and mildly symptomatic infected pregnant women outnumber those with severe symptoms. Current studies, however, focus on severe cases that required hospitalizations. These studies examine maternal and perinatal death rates, vertical transmission from mother to fetus, and obstetric and neonatal outcomes (3–7). There remains a gap in knowledge of the impact of the infection in a majority of asymptomatic or mildly symptomatic women. A recent review reported a high rate of elective preterm cesarean delivery (8). Maternal and fetal health throughout the trimesters should be examined carefully regardless of severity of symptoms. More evidence is required to guide obstetric practices.

Hoffmann et al. elucidated the mechanism of host cell entry of SARS-CoV-2. They determined that angiotensin converting enzyme 2 (ACE2) is the receptor that allows the binding of SARS-CoV-2 spike proteins and, through this binding process, enters host cells (9). The importance of ACE2 in SARS-CoV infection was established in the early 2000s. Li et al. first isolated the protein in SARS-CoV permissive cells and showed that ACE-2 antibodies blocked viral replication (10). A year later, a research group showed the correlation between susceptibility to SARS-CoV infection and the level of expression of ACE2 *in vitro* (11). The study supports the hypothesis that a higher expression of ACE2 leads to higher risk of SARS-CoV-2 infection. Furthermore, studies have shown that SARS-CoV infections downregulate ACE2 expression and promotes more severe disease progression (12–14). ACE2 is expressed in many organs including lung, stomach, kidney, heart, brain, and reproductive tissues (15, 16). Theoretically, once the virus establishes its primary infection through the respiratory system, it can spread to any other organs expressing ACE2 through the bloodstream and downregulate the local expression of ACE2. Studies investigating SARS-CoV-2 infection other than the respiratory system have started to emerge (17, 18). The public health implications of the spread of SARS-CoV-2 infection from the initial site of infection to the female reproductive organs, in both pregnant and non-pregnant reproductive age women, are the focus of this paper.

## SARS-CoV-2 Infection in Pregnancy and Maternal Health

Asymptomatic and mildly symptomatic pregnant women face two unique risks posed by SARS-CoV-2 infection due to changes in ACE2 expression to accommodate hemodynamic changes in pregnancy. The first risk involves the increased expression and activity of ACE2 during pregnancy and possible secondary uteroplacental infection. Increased expression of ACE2 during pregnancy was suggested by an animal study to play a functional role in maintaining a normal blood pressure despite an increase in plasma volume of 20–70% toward the end of pregnancy (16). ACE2 has been shown to metabolize angiotensin II (Ang II) to Ang-(1-7) (19). Ang II constricts blood vessels while Ang-(1-7) dilates vessels. Relative expression of Ang II and Ang-(1-7), heavily influenced by ACE2 expression, was proposed to maintain normal blood pressure. Another study confirmed this finding (19). The study compared mean blood pressure (MAP) and plasma Ang-(1-7) levels in ACE2 knockout (KO) and wild type (WT) pregnant mice and found a statistically significant increase in both MAP and plasma Ang (1-7) level in ACE2 KO mice (20). The same study also found an association between ACE2 deficiency and an impaired maternal gestational weight (20). The uterus and the placenta, with their enhanced expression and activity of ACE2 during pregnancy (16), may put pregnant women at an increased risk of establishing a secondary uteroplacental SARS-CoV-2 infection.

The second risk involves downregulation of ACE2 by the SARS-CoV-2 virus in pregnancy (14). In the study discussed above, in which the authors compared MAP and plasma Ang-(1-7) levels in ACE2 KO and WT pregnant mice, a statistically significant increase in both MAP and plasma Ang (1-7) level in ACE2 KO mice was found (20). This finding suggests another potential risk for the mothers, the potential risk of developing preeclampsia.

To evaluate the first risk of a secondary uteroplacental infection, studies should address the relationship between level of ACE2 expression in placenta and uterine tissues and tissue susceptibility to SARS-CoV-2 viral invasion. The second risk of developing preeclampsia needs to be assessed through human studies comparing the percentage of preeclamptic women in SARS-CoV-2 positive patients with preeclampsia in non-COVID-19 patients. A recent article suggested that a high plasma soluble ACE2 level might be protective for SARS-CoV-2 infection (21). The authors explained that this paradoxical observation might be due to the posttranslational events regulating protein levels and a balance between soluble and membrane-bound form. These results will need additional confirmation. Normal hemodynamics in placenta may be affected by viral-induced downregulation of ACE2 expression in COVID-19 pregnant women. A study showed a higher Ang II in placenta in ACE2 KO mice compared to WT mice (20). Previously, an increase in Ang II in both the maternal and fetal components of the placenta has been found in human transgenic rat model of preeclampsia (22). This increase in Ang II was also observed in the chorionic villi of the placenta of women with preeclampsia (23). The increased level of Ang II in placenta could lead to placental ischemia. A recent study of three women with confirmed SARS-CoV-2 infection who delivered by cesarean delivery described placental pathology (24). All three women had fever, one before delivery and the other two postpartum. Samples taken from the three placentas were negative for nucleic acid of SARS-CoV-2. Various degrees of fibrin deposition inside and in proximity to villi as well as local syncytial nodule increases were observed in all three placentas. One displayed massive placental infarction. No pathological placental changes due to SARS-CoV-2 was found in the three placentas. Such morphological studies of placenta should be extended to mildly symptomatic and asymptomatic women. To understand if the infection compromises blood flow to the placenta, placental tissue samples need to be collected from SARS-CoV-2 infected and healthy women. These samples should be evaluated for signs of ischemia. Furthermore, the use of ultrasound may demonstrate evidence of placental vascular compromise. A possible correlation between SARS-CoV-2 infection and incidence of placental ischemia needs to be understood through such studies.

In addition, whether there is a progressive increase in risk throughout the trimesters in developing cardiovascular and respiratory insufficiency should be addressed. Current published clinical experience is limited to women who developed symptoms in late third trimester and were delivered shortly after the diagnosis (8).

## SARS-CoV-2 Infection in Pregnancy and Fetal Health

In mildly and asymptomatic COVID-19 pregnant women, the dysregulation of ACE2-Ang-(1-7) and its receptor MasR axis may have implications for the fetus. This could occur at both the intrapartum period and long-term. A study in rats showed that maternal glucocorticoid treatment reduced levels of ACE2 and Ang-(1-7) in rat placenta, specifically in fetal part labyrinth zone where nutrient and waste exchange occurs in late pregnancy (25). The animal study further correlated this reduction in ACE2 and Ang-(1-7) with impaired intrauterine fetal growth. A recent review of both animal and human studies suggested that alterations in Ang-(1-7) axis, particularly within the kidney and brain during perinatal programming, could lead to increased risk of development of hypertension and cardiovascular disease (26). To assess if the infection is associated with restricted intrauterine fetal growth, studies need to monitor ACE2, Ang-(1-7) levels and the growth of fetus in both infected and healthy women. To evaluate if fetus born to infected mothers are at a higher risk of developing hypertension and cardiovascular diseases later in life compared to fetus born to healthy mothers, the infants need to be monitored for blood pressure and cardiovascular abnormalities into early and late adulthood.

## SARS-CoV-2 Infection and Reproductive Health

Mildly symptomatic and asymptomatic COVID-19 women may have issues in planning for future reproduction. In particular, three issues, sexual transmission of the virus, the use of contraceptives and the risk of infertility, may be public health concerns. Recent studies have shown that no SARS-CoV-2 virus was detected in vaginal fluids or semen in infected individuals (27, 28). The sample size of both studies, however, is small. Larger-scale studies remain to be conducted to confirm this finding. Nevertheless, there is currently no evidence supporting sexual transmission of the virus. The usage of contraception should be examined for patients with an ongoing infection. For SARS-CoV-2 patients with an ongoing infection, whether estrogen and progesterone contained in contraceptive agents alters ACE2 expression and induces pregnancy-like risks is unclear. The dose-response effect of hormones contained in contraceptive agents on uterine and endometrial ACE2 expression should be studied. In addition, studies should address whether SARS-CoV-2 infections affect the overall efficacy of contraception, whether the contraception is via steroid hormones or by intrauterine devices.

COVID-19 infections may have implications for infertility patients. The enhanced expression of ACE2 in the placenta and uterus during early pregnancy (16) after infertility treatment may make the organs more susceptible to viral entry during mild or asymptomatic infections. The local placenta-uterus infection could induce inflammation and subsequent scarring that may compromise future fertility. In addition, ACE2 is found in human ovarian follicles and the endometrium (29, 30). As a consequence, patients with mild or asymptomatic SARS-CoV-2 infections may have difficulty with their ovarian ovulation induction protocols or with implantation of embryos in the endometrium. Among patients without previous history of infertility, whether mild or asymptomatic SARS-CoV-2 infections increases the infertility rates should be examined. Thus, for infertility, studies could include an examination of whether a secondary infection occurs at a higher rate in mild or asymptomatic infections, whether the infection affects ovulation induction or embryo implantation and whether a more general increase in rates of infertility is found in these patients.

## Discussion

The health risks of mild and asymptomatic SARS-CoV-2 infected women face in pregnancy have not been investigated in detail. As of May 2020, there have been more than 5 million confirmed cases worldwide with daily confirmed cases around 90,000^1^^,^^2^. Assuming that 80% of confirmed cases are asymptomatic and mildly symptomatic as recent reports suggested (31), there are already close to four million people worldwide in this category. There is an urgent need to understand what these risks might be and prepare families for current and future pregnancy challenges. So far, there have been no conclusive evidence on how the infection differentially affects pregnant women. They are in fact advised to take the same precautions as the general public. Public health initiatives should identify pregnant women infected with Covid-19 with antibody tests and monitor maternal and fetal health closely. Initiatives should also include non-pregnant women to be monitored for future reproductive issues.

There is a theoretical public health concern for SARS-CoV-2 to impact both a current pregnancy and future reproduction in mildly symptomatic and asymptomatic women. This theoretical issue is rooted in two findings that have been substantiated by several studies. The first is the association between the SARS-CoV-2 infection and disrupted ACE2 expression. Disrupted ACE2 expression is likely to lead to dysregulation in ACE2 Ang-(1-7) axis. The other finding is an association between dysregulated ACE2 Ang-(1-7) axis and impaired maternal and fetal health. Combining the two findings, we hypothesize that SARS-CoV-2 impairs cardiovascular adaptation of mothers, normal hemodynamic regulation of placenta, fetal growth and long-term cardiovascular health, as well as reproductive health of women in general.

The significance of ACE2 in the infection of SARS-CoV-2 is clear. Current evidences are mainly derived from animal studies. Future studies may consider distinguishing the function of soluble and membrane-bound form of ACE2 in viral invasion, early and late stages of the infection. Regardless of whether soluble and membrane bound forms of ACE2 play distinct roles, we think that ACE2 Ang-(1-7) axis is disrupted to different degrees during the infection. And this becomes a critical question that needs to be addressed in future studies.

As studies continue to shed light on the effect of the infection on different organ systems, they should engage the research community, clinicians, and the public to reassess the impact of the infection on reproduction. Based on evidence discussed here, we think that there are effects on mothers, placenta, and the fetus throughout the trimesters. In addition, we hypothesize that these effects may extend into future reproduction of mildly symptomatic and asymptomatic COVID-19 women. Given the risks associated with asymptomatic SARS-CoV-2 infections as well as inconclusive evidence surrounding vertical transmission, women of reproductive age may need to be advised about the theoretical risks to the mother and fetus until more is known.

## Author Contributions

All authors listed have made a substantial, direct and intellectual contribution to the work, and approved it for publication.

## Conflict of Interest

The authors declare that the research was conducted in the absence of any commercial or financial relationships that could be construed as a potential conflict of interest.

## Footnotes

^1^COVID-19 situation update worldwide, as of 6 May 2020. Available at: https://www.ecdc.europa.eu/en/geographical-distribution-2019-ncov-cases (accessed May 6, 2020).

^2^Daily confirmed COVID-19 cases. Available at: https://ourworldindata.org/grapher/daily-cases-covid-19 (accessed May 6, 2020).
